# Supplementary material for: Complexome profiling of the Chlamydomonas psb28 mutant reveals TEF5 as an early PSII assembly factor
Source: Plant Cell. 2025 Mar 17;37(6):koaf055. doi: 10.1093/plcell/koaf055 (PMC12164590; doi:10.1093/plcell/koaf055)
Supplement: koaf055_Supplementary_Data [file koaf055_supplementary_data.zip › psb28_interactive.zip!/plots/intensity/qualityPlots/peptides/_Cre12.g553678.t1.1.html]

### Cre12.g553678.t1.1

Trivial name:   
SeqCoverage [median]: 18.80  
Mass: 12.13 kDa  
  
 

| Sequence | MissedCleavage | Mass [Da] | wtIntensity | muIntensity | Score |
| --- | --- | --- | --- | --- | --- |
| ALTLPGTGLK | 0 | 969.59 | 2058.37 | 0.00 | 84.57 |
| ALTLPGTGLKR | 1 | 1125.69 | 6768.81 | 5295.88 | 78.69 |
| EAIELGNSLAK | 0 | 1143.61 | 237.58 | 1418.19 | 127.40 |
|  |  |  | 9064.8 | 6714.1 |  |
